# Supplementary material for: Reversible HuR‐microRNA binding controls extracellular export of miR‐122 and augments stress response
Source: EMBO Rep. 2016 Jul 11;17(8):1184–203. doi: 10.15252/embr.201541930 (PMC4967961; doi:10.15252/embr.201541930)
Supplement: Supplementary file 1 — Expanded View Figures PDF [file EMBR-17-1184-s001.pdf]

Expanded View Figures

Figure EV1. Characterization of EVs released by Huh7 cells.

A Real-time qRT–PCR was done and mean C_t values of RNA isolated from EVs isolated from different types of cell culture media. Same amounts of DMEM medium either containing normal FCS or with commercial exosome-free FCS (before and after the growth of Huh7 cells) were used for EV isolation and subsequent miR-122 estimation (mean ± s.e.m., n = 3).

B Nanoparticle tracking analysis (NTA) of EVs isolated by ultracentrifugation from Huh7 cell. Size and particle distribution plots of isolated EVs. The FTLA size distribution of the vesicles is shown, and red bars indicate standard errors of mean.

C Immobilized biotinylated antibodies against the tetraspanins/CD63 on magnetic streptavidin beads were used to pull down respective protein-containing vesicles from isolated Huh7 EVs. Exo-FITC universal exosomes stain in the same mix enabled the EVs to be visualized by FACS. In the presence of Huh7 EVs, FITC fluorescence shift indicated presence of the biochemical markers on the exosomes.

D Tapping mode amplitude and phase AFM images showing round morphology of isolated EVs.

E Effect of α-amanitin on miR-122 and CAT-1 mRNA levels in Fed and Starved Huh7 cells pre-treated with GW4869. Cellular miRNA and mRNA levels after 4 h of α-amanitin treatment were normalized against U6 snRNA and GAPDH mRNA, respectively (mean ± s.e.m., n = 3).

F Snapshot of TUNEL-positive cells (green) detected in Fed and Starved Huh7 cell population. Nuclei of individual cells were stained with DAPI. DNase I-treated cells were used as positive control. Scale bar, 50 μm.

Data information: ns: non-significant, *P < 0.05. P-values were determined by paired t-test. For estimations of relative level of either miRNAs or mRNAs, C_t values only within the range of 20–32 were considered for analysis.

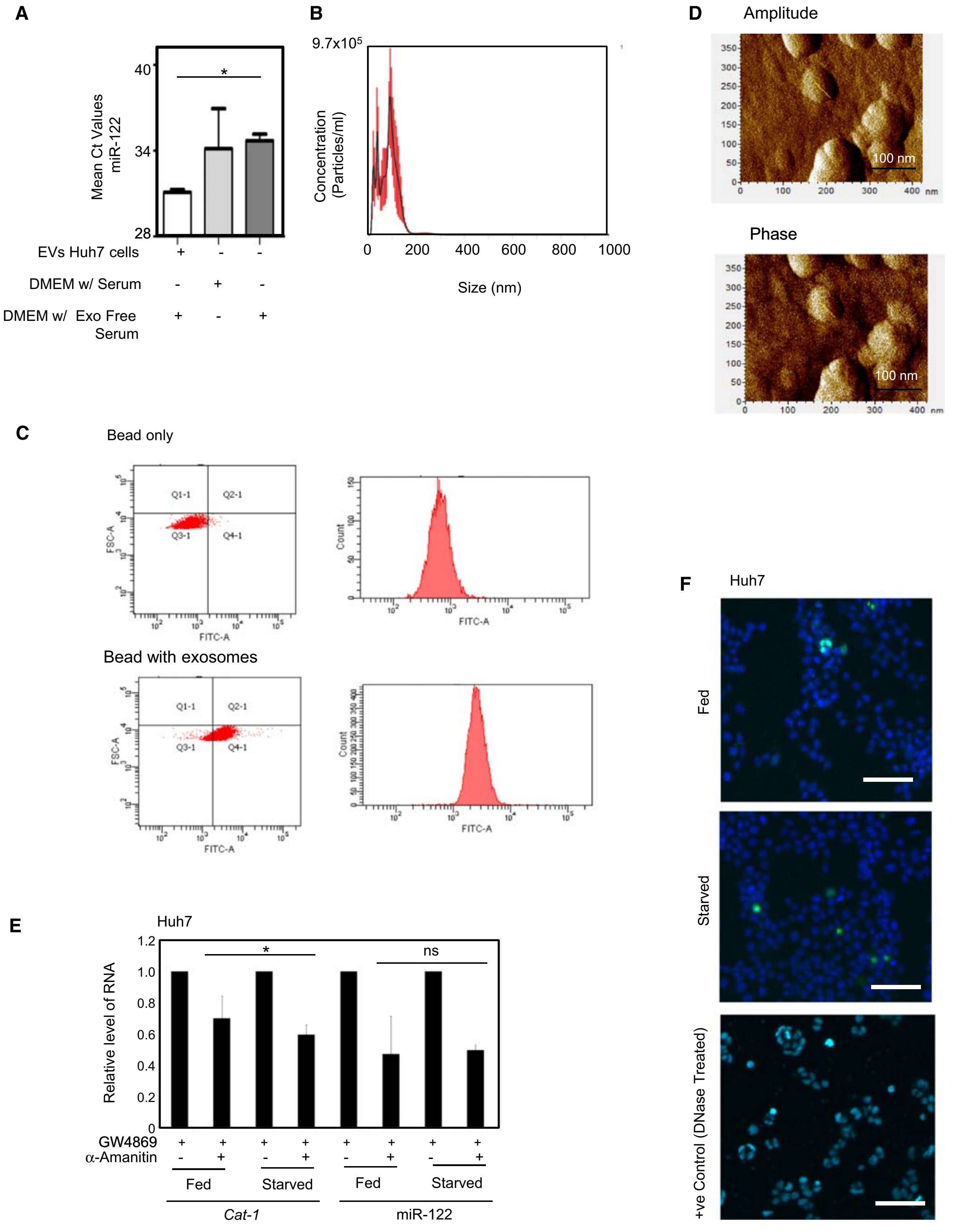

Figure EV1.

 © 2016 The Authors

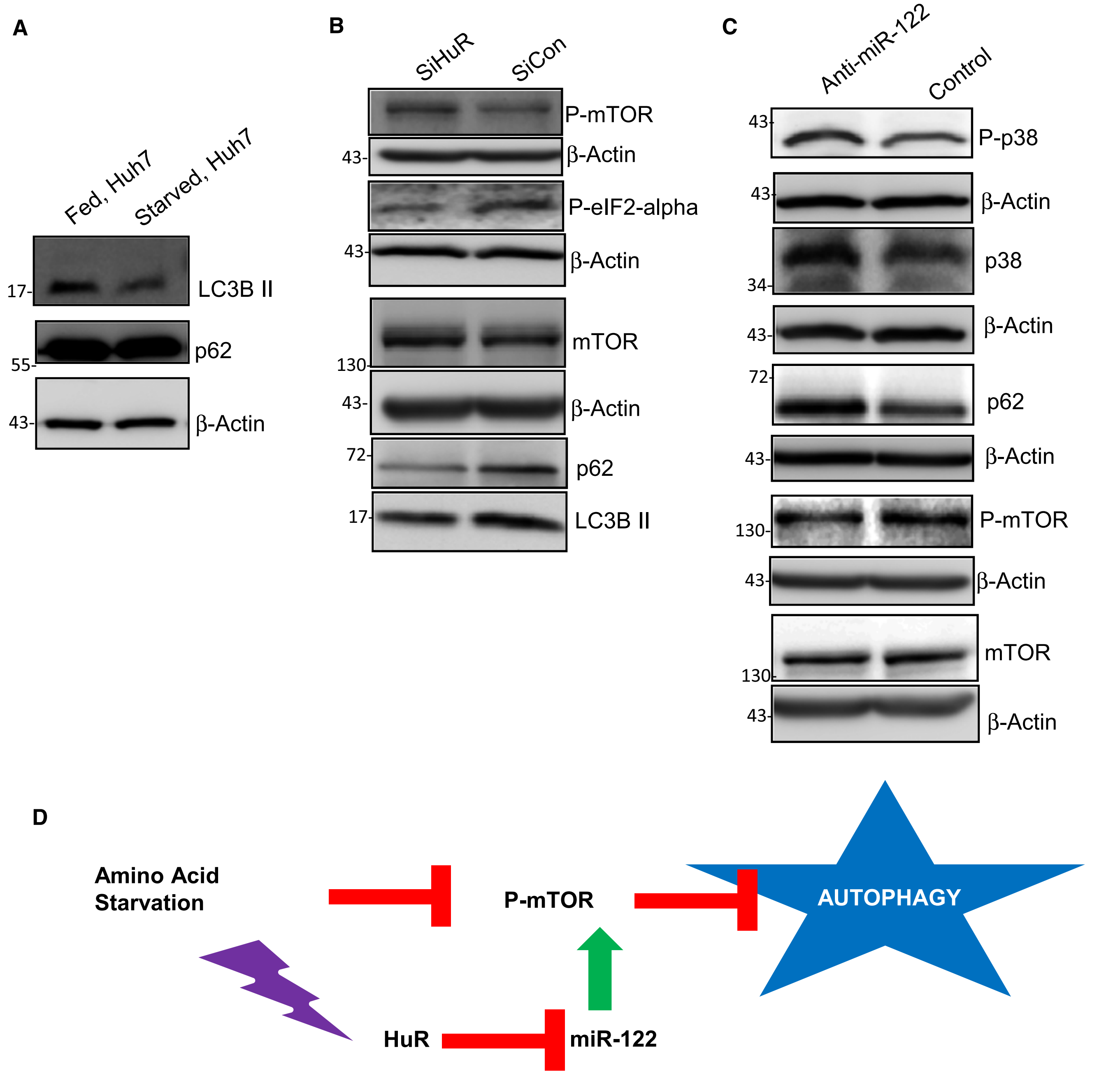

Figure EV2. Effect of HuR depletion and miR-122 inactivation on mTOR and p38 phosphorylation and expression of autophagy markers.

A Cell lysates of Fed and Starved Huh7 cells were immunoblotted for the autophagy marker proteins LC3BII and p62.

B Effect of HuR depletion on mTOR and its phosphorylated form in Huh7 cells. Levels of p62 and LC3BII were also detected.

C Effect of anti-miR-122 or control oligonucleotides on the levels of p38 and mTOR and their phosphorylated forms in HuR-depleted Huh7 cells Starved for 8 h. Levels of p62 were also measured.

D Schematic representation of regulation of autophagy in Huh7 cells by HuR and miR-122.

Data information: Positions of size markers in protein gels used for respective Western blot analysis are shown against each panel. In all cases, β-actin served as loading control.

© 2016 The Authors

Figure EV3. HuR-driven EV-mediated export of miRNA let-7a in MDA-MB-231 cells controls cell proliferation and senescence.

A Effect of siRNA-mediated HuR depletion on cellular and EV-associated let-7a level. Control siRNA (SiCon)-treated cells were used as reference. miRNA levels were analyzed by qRT–PCR (mean ± s.e.m., n = 5). U6 snRNA levels were used for normalization of cellular miRNA content.

B, C Effect of GW4869 treatment on senescence status of MDA-MB-231 cells. Representative pictures of senescence status of MDA-MB-231 are shown in (B), while the quantification (left panel) and expression status of few senescence-related proteins (right panel) are shown in (C) (mean ± s.e.m., n = 3).

D, E Effect of inactivation of let-7a on senescence status of MDA-MB-231. Growth-retarded MDA-MB-231 cells were treated with either anti-let-7a or anti-miR-122 oligonucleotides (control) and senescence status was measured (mean ± s.e.m., n = 5).

F Effect of let-7a expression on proliferation status of MDA-MB-231 cells treated or untreated with exosomal export blocker GW4869. Nuclei were stained for PCNA and percentage of cells with PCNA-positive nuclei were calculated and plotted (mean ± s.e.m., n = 3).

G Effect of HuR depletion on the let-7a-induced senescence. Senescence levels of siControl- or siHuR-treated MDA-MB-231 cells pre-transfected with pre-let-7a RNA were measured and plotted (mean ± s.e.m., n = 3).

H–J Effect of Myc-HuR expression on the HCV replicon RNA level and cellular miR-122 content in Huh7 cells. A possible model of HCV replication regulation by miR-122 and HuR (H). Relative change in expression level of miR-122 (I) and HCV replicon RNA (J) upon co-transfection with Myc-HuR. The expression of Myc-HuR was confirmed by Western blot for HuR in (J) and marked by arrow.

Data information: Positions of size markers in protein gels used for respective Western blot analysis are shown against each panel. *$P < 0.05$, **$P < 0.01$, ***$P < 0.0001$. P-values were determined by paired t-test. For estimations of relative level of either miRNAs or viral RNAs, C_t values only within the range of 20–32 were considered for analysis.

 © 2016 The Authors

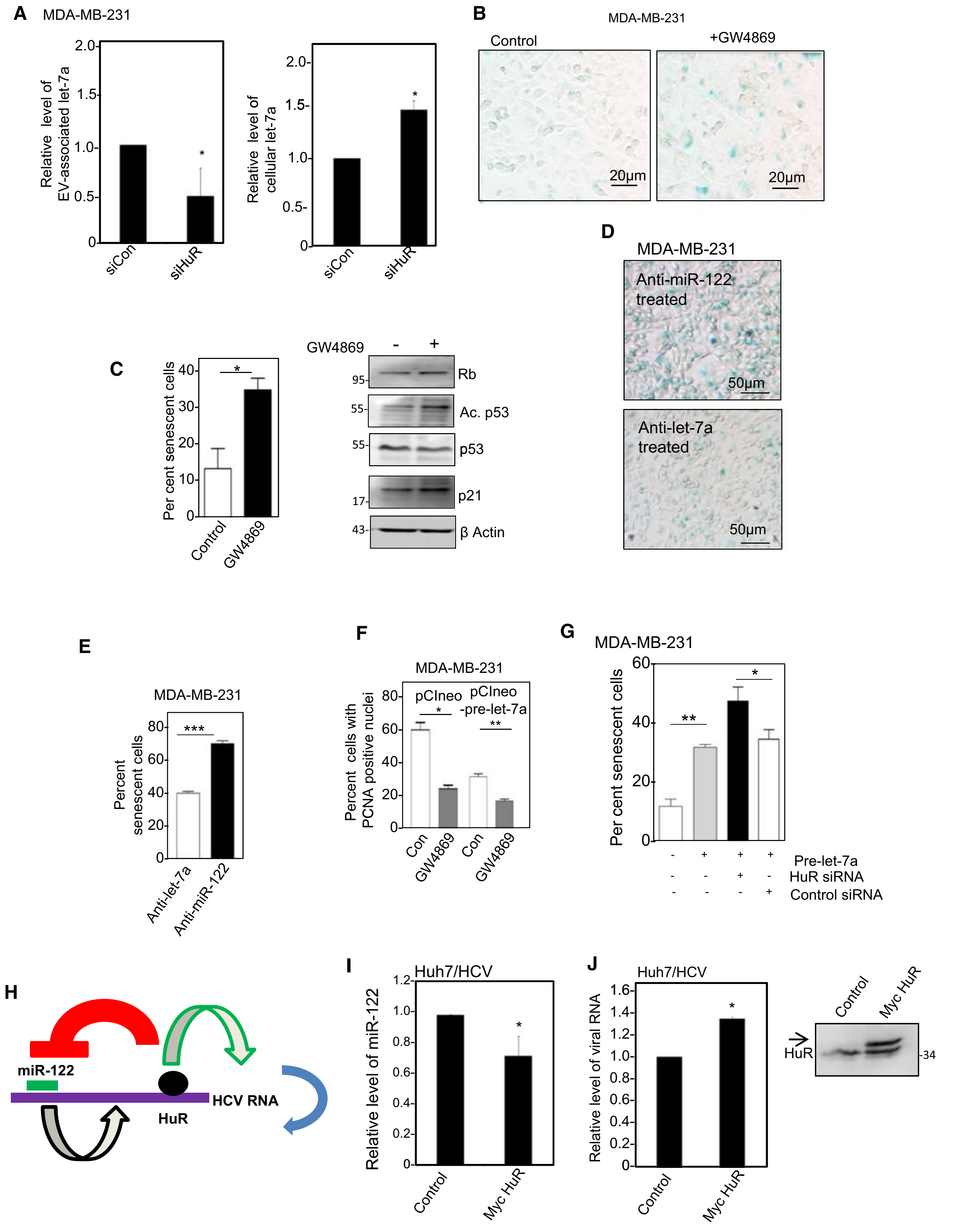
A
MDA-MB-231
Relative level of EV-associated let-7a
Relative level of cellular let-7a
siCon
siHuR
B
MDA-MB-231
Control
+GW4869
20μm
C
Per cent senescent cells
Control
GW4869
GW4869 - +
Rb
Ac. p53
p53
p21
β Actin
D
MDA-MB-231
Anti-miR-122 treated
Anti-let-7a treated
50μm
E
MDA-MB-231
Percent senescent cells
Anti-let-7a
Anti-miR-122
F
MDA-MB-231
Percent cells with PCNA positive nuclei
pCIneo
pCIneo -pre-let-7a
Con
GW4869
G
MDA-MB-231
Per cent senescent cells
Pre-let-7a
HuR siRNA
Control siRNA
H
miR-122
HCV RNA
HuR
I
Huh7/HCV
Relative level of miR-122
Control
Myc HuR
J
Huh7/HCV
Relative level of viral RNA
Control
Myc HuR
HuR

Figure EV3.

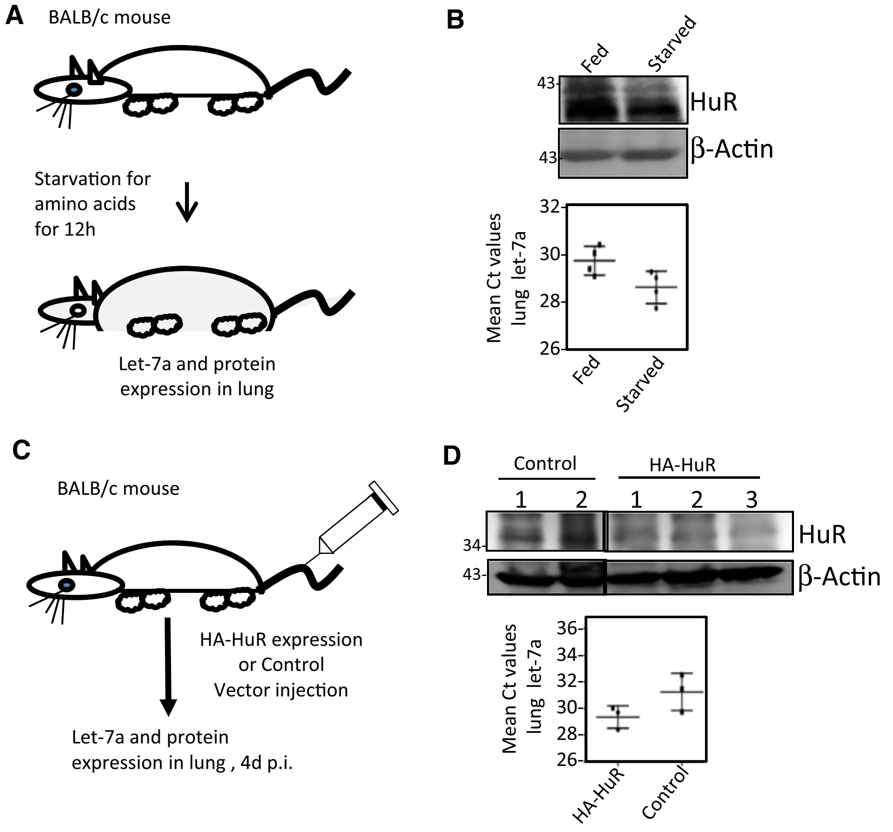

Figure EV4. Effect of starvation and exogenous HA-HuR expression on lung HuR and miRNA levels in BALB/c.

A Scheme of the starvation experiment is shown.

B Effect of starvation on lung HuR and let-7a miRNA levels in the lungs of Starved animals determined by Western blot and qRT–PCR, respectively. Mean C_t values for RNA samples isolated from the lungs of Fed and Starved animals were shown (mean ± s.e.m., n = 4). β-Actin was used as loading control in HuR Western blot.

C Scheme of the experiment where HA-HuR expression or control (pCIneo) plasmids were injected via tail vein.

D Effect of HA-HuR expression plasmid injection on lung HuR level in mouse (mean ± s.e.m., n = 3). Western blot was done on lung lysates obtained from control or HA-HuR plasmid-injected animals. β-Actin was used as loading control. Relative levels of let-7a miRNA are represented as mean C_t values for the same amount of lung RNA used.

Data information: Positions of size markers in protein gels used for respective Western blot analysis are shown against each panel. p.i.: post injection.

Figure EV5. HuR binding replaces Ago2 from target mRNAs.

A, B miRNP and HuR binding to common target message is mutually exclusive. Association of CAT-1 and a miR-122 reporter RL-catA mRNAs with HuR and HA-Ago2 in control (Fed) and Starved (Starved) Huh7 cells expressing HA-Ago2 along with RL-catA (having both HuR and miR-122-binding sites) or RL-con reporters (without miR-122 and HuR-binding sites). A scheme of the experiment is shown in the upper panel. HA-Ago2 and HuR were immunoprecipitated with anti-HA or anti-HuR-specific antibodies, respectively, from both Fed and Starved cell lysates, and associated mRNAs were detected by semi-quantitative RT–PCR (lower panel). The Western blot data are shown in (B). Anti-GFP antibody was used as control in immunoprecipitation.

C Association of CAT-1, aldolase, and GAPDH mRNAs with Ago2 in Huh7 cells co-expressing either control or HA-HuR-encoding plasmid along with FH-Ago2-encoding plasmid. Real-time estimation of Ago2-associated mRNA was normalized against immunopurified FH-Ago2 level, and estimation was done from three independent sets (mean ± s.e.m., n = 3).

D Effect of FH-Ago2 expression on cellular and exosomal miR-122 levels in HA-HuR-expressing Huh7 cells (middle panels) (mean ± s.e.m., n = 3). The expression levels of Ago2 and HuR in cells transfected with HA-HuR and FH-Ago2 expression plasmids are shown in the upper panel. Effect of Ago2 expression on cellular miR-122 levels in Starved Huh7 cells (lower panel). Ago2 expression levels were detected by Western blot.

E RNA gel shift assay done with ^{32}P-end labeled TNF-α AU-rich sequence containing HuR-binding substrate and recombinant full-length or the truncated version HuR-ΔIII. The position of gel shifted bands after forming the complexes are marked by arrowheads. Positions of the free probe are marked by arrows.

F RNA gel shift assay done with 10 nM of ^{32}P-end labeled miR-122 and miR-122* RNA and with increasing concentrations of recombinant full-length HuR. The position of gel shifted bands after forming complexes is marked by arrowheads. Positions of the free probes are marked by arrows. The ^{32}P-end labeled miR-122 and miR-122* hybrid is marked by #.

Data information: Positions of size markers in protein gels used for respective Western blot analysis are shown against each panel. ns: non-significant, *P < 0.05, **P < 0.01, P-values were determined by paired t-test. For estimations of relative level of either miRNAs or mRNAs, C_t values only within the range of 20–32 were considered for analysis.

▶

 © 2016 The Authors

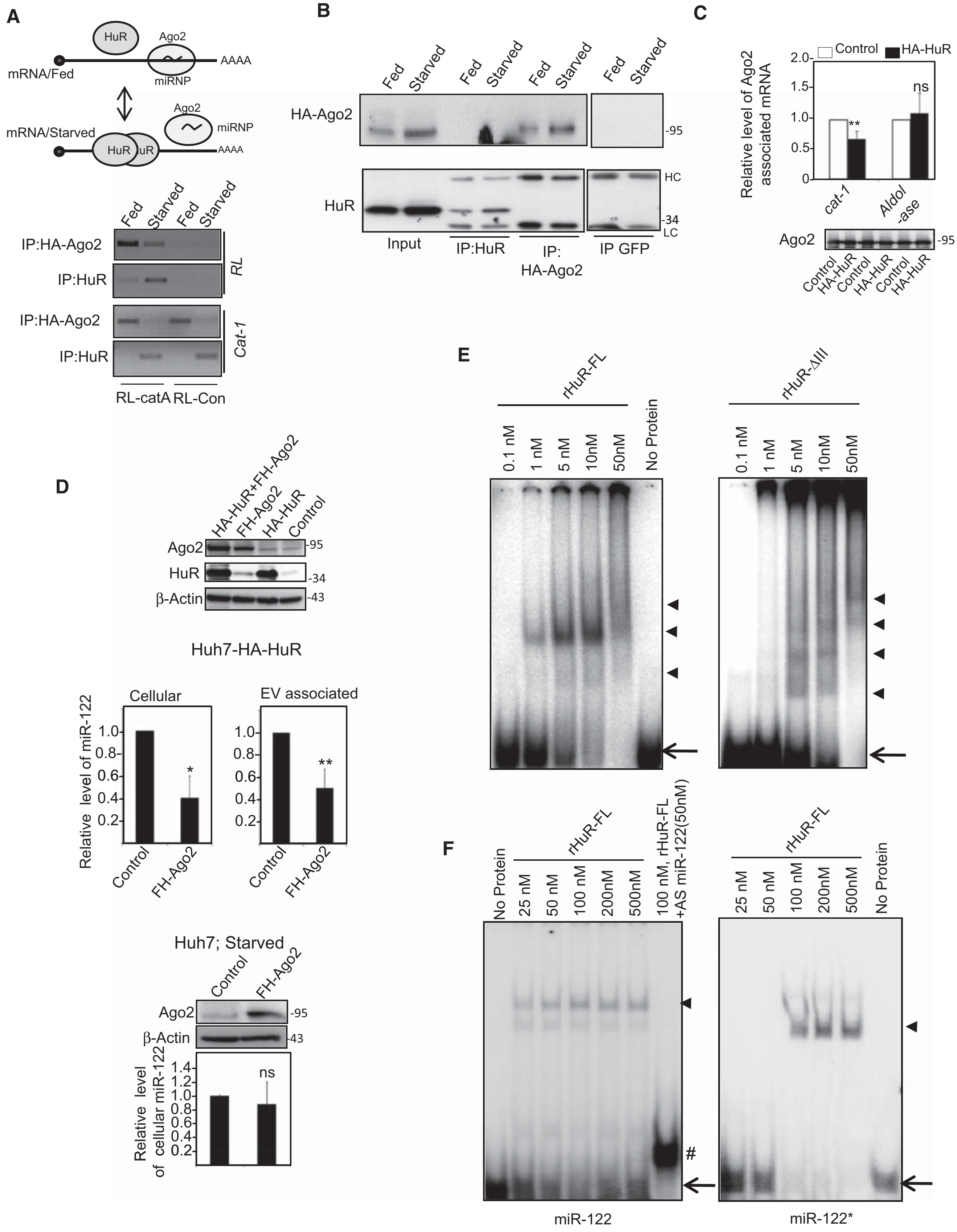
A
HuR
Ago2
AAAA
mRNA/Fed
miRNP
Ago2
miRNP
mRNA/Starved
HuR
AAAA
Fed Starved Fed Starved
IP:HA-Ago2
IP:HuR
RL
IP:HA-Ago2
IP:HuR
Cat-1
RL-catA RL-Con
B
Fed Starved Fed Starved Fed Starved Fed Starved
HA-Ago2
-95
HuR
HC
-34
LC
Input
IP:HuR
IP: HA-Ago2
IP GFP
C
Control HA-HuR
Relative level of Ago2 associated mRNA
ns
**
cat-1
Aldol -ase
Ago2
-95
Control HA-HuR Control HA-HuR Control HA-HuR
D
HA-HuR+FH-Ago2 FH-Ago2 HA-HuR Control
Ago2 -95
HuR -34
β-Actin -43
Huh7-HA-HuR
Cellular
EV associated
Relative level of miR-122
Control FH-Ago2
Huh7; Starved
Control FH-Ago2
Ago2 -95
β-Actin -43
Relative level of cellular miR-122
ns
E
rHuR-FL
0.1 nM 1 nM 5 nM 10nM 50nM No Protein
rHuR-ΔIII
0.1 nM 1 nM 5 nM 10nM 50nM
F
rHuR-FL
No Protein 25 nM 50 nM 100 nM 200nM 500nM 100 nM, rHuR-FL +AS miR-122(50nM)
#
miR-122
rHuR-FL
25 nM 50 nM 100 nM 200nM 500nM No Protein
miR-122*

Figure EV5.
