# Supplementary material for: Reversible HuR‐microRNA binding controls extracellular export of miR‐122 and augments stress response
Source: EMBO Rep. 2016 Jul 11;17(8):1184–203. doi: 10.15252/embr.201541930 (PMC4967961; doi:10.15252/embr.201541930)
Supplement: Supplementary file 2 — Table EV1 [file EMBR-17-1184-s002.doc]

**Table EV1** List of Plasmids and siRNAs

| **Name of Plasmid** | **Reference/Source** | **Plasmid Description** |
| --- | --- | --- |
| pRL-Con | From Witold Filipowicz | Humanized Renilla Luciferase coding region. |
| FLAG-HA-AGO2 | From Tom Tuschl | Expressing FLAG & HA tagged human AGO2 |
| pmiR-122 |  | Plasmid encoding pre-miR-122 under a constitutive U6 promoter |
| FLAG-HuR, HA-HuR and HA-HuR∆H | From Witold Filipowicz | Full length and truncated HuR without hinge region (HNS) with HA coding sequence in pCIneo vector |
| pRK5-HA-Ubiquitin-WT | From Partha Chakrabarty | Encoding the HA-tagged version of human Ubiquitin |
| pcDNA3-myc-HuR | - | HuR coding sequence between the EcoRI and XhoI sites of pcDNA3.1 containing the myc tag sequence between BamHI and EcoRI sites |
| pHCV-JFH1 | From Takaji Wakita | Encoding Infectious HCV-JFH1 |
| pPre-let-7a | - | Encoding the coding and promoter element of human let-7a in pCIneo vector. |
| siRNA (Neutral Sphingomyelinase 2) SMPD 2 | Dharmacon | ON-TARGETplus SMARTpool - Human SMPD2 |
| si All Star Negative (siCon) | Qiagen | Negative control siRNA |
| si RNA HuR | Dharmacon | | ON-TARGETplus SMARTpool - Human ELAVL1  ON-TARGETplus SMARTpool Mouse elavl1 | | --- | |
| SYNTHETIC  MiR-122 (5p) | Eurogentec | 5’UGGAGUGUGACAAUGGUGUUUG3’ |
| TNFα 3‘UTR | IDT | 5’ AUUAUUUAUUAUUUAUUUAUUAUUUAUUUAUUUA |
| miR-122 target synthetic RNA sequence | IDT | 5’AAAUUCAAACACCAUUGUCACACUCCACCAGAUUAA3’ |
