# Supplementary material for: Reversible HuR‐microRNA binding controls extracellular export of miR‐122 and augments stress response
Source: EMBO Rep. 2016 Jul 11;17(8):1184–203. doi: 10.15252/embr.201541930 (PMC4967961; doi:10.15252/embr.201541930)
Supplement: Supplementary file 3 — Table EV2 [file EMBR-17-1184-s003.doc]

**Table EV**2 List of primers used

| **Target** | **Forward Primer** | **Reverse Primer** |
| --- | --- | --- |
| [ALDOLASE](http://www.ncbi.nlm.nih.gov/entrez/viewer.fcgi?db=nucleotide&id=342187192) | 5' TGGACCTAGCTTGGCGCGGA 3' | 5' CCTGGGCCAGCAGGCAGTTC 3' |
| GYS1 | 5' GGTGGCTAACAAGGTGGGTGGC 3' | 5' CGATCAGCCAGCGCCCGAAA 3' |
| CAT1 | 5' GCCGCCGGCTTGGATTCTGA 3' | 5' CCCCGAGGGCCACCAGATCA 3' |
| 18S rRNA | 5' TGACTCTAGATAACCTCGGG 3' | 5' GACTCATTCCAATTACAGGG 3' |
| GAPDH | 5‘ AAAAGCGGGAGAAAGTAGG 3‘ | 5‘ AAGAAGATGCGGCTGACTGT 3‘ |
| Pre- miR 122 | 5‘ AGCTGTGGAGTGTGACAATG 3‘ | 5‘ GCTATTTAGTGTGATAATGGCG 3‘ |
| HMGA2/5'UTR | 5‘ CTTGAATCTTGGGGCAGG 3‘ (18) | 5‘ CCTGCCTCCCGCCGCC 3‘ (16) |
