# Supplementary material for: Reversible HuR‐microRNA binding controls extracellular export of miR‐122 and augments stress response
Source: EMBO Rep. 2016 Jul 11;17(8):1184–203. doi: 10.15252/embr.201541930 (PMC4967961; doi:10.15252/embr.201541930)
Supplement: Supplementary file 4 — Table EV3 [file EMBR-17-1184-s004.doc]

**Table EV3 List of Antibodies used**

| **Name of Antigens** | **Raised in** | **Source** | **Dilutions used for Western Blot** | **Catalog No.** |
| --- | --- | --- | --- | --- |
| β-Actin | Mouse Monoclonal (HRP conjugated) | Sigma Aldrich | 1:10000 | A3854 |
| HA | Rat Monoclonal  ( clone 3F10) | Roche | 1:1000 | 11 867 423 001 |
| AGO2 (eIF2C2) | Mouse Monoclonal | Novus Biologicals | 1:500 | H00027161-M01 |
| RCK/p54 | Rabbit | Bethyl Laboratories | 1:10000 | A300-461A |
| HRS | Rabbit | Bethyl Laboratories | 1:1000 | A300-989A |
| β Tubulin | Mouse | Sigma | 1:1000 | T5201 |
| Calnexin | Rabbit | Cell Signalling Technology | 1:1000 | #2679S |
| Alix | Mouse Monoclonal | Santa Cruz | 1:500 | sc53538 |
| HuR | Mouse Monoclonal  (clone 3A2) | Santa Cruz | 1:1000 | sc-5261 |
| Phospho 4E-BP1 | Rabbit | Cell Signalling Technology | 1:500 | #2855 |
| eIF2 alpha | Rabbit | Cell Signalling Technology | 1:500 | #9722 |
| Phospho eIF2 alpha | Rabbit | Cell Signalling Technology | 1:500 | #9721 |
| Cytochrome C | Rabbit | Cell Signalling Technology | 1:1000 | #4272 |
| LC3B | Rabbit | Cell Signalling Technology | 1:500 | #3868P |
| P 62 | Rabbit | BioBharati LifeScience | 1:1000 | BB-AB0130 |
| P38 | Rabbit | Cell Signalling Technology | 1:1000 | #9212P |
| Phospho P38 | Rabbit | Cell Signalling Technology | 1:1000 | #4511P |
| mTOR | Rabbit | Cell Signalling Technology | 1:1000 | #2983S |
| Phospho mTOR | Rabbit | Cell Signalling Technology | 1:1000 | #2971S |
| HSP90 | Rabbit | Cell Signalling Technology | 1:1000 | #4877 |
| CD63 | Mouse | BD Pharmingen | 1:500 | 556019 |
| Ubiquitin | Rabbit | Bethyl Laboratories | 1:1000 | A300-317A |
| GAPDH | Mouse | Sigma | 1:1000 | G8795 |
| GFP | IgG control | Roche | 1:1000 | 11 814 460 001 |
| 6-HIS | Rabbit | Bethyl | 1:1000 | A190-114A |
