# Supplementary figures and images for: Reversible HuR‐microRNA binding controls extracellular export of miR‐122 and augments stress response
Source: EMBO Rep. 2016 Jul 11;17(8):1184–203. doi: 10.15252/embr.201541930 (PMC4967961; doi:10.15252/embr.201541930)

**Source Data Figure 1A**

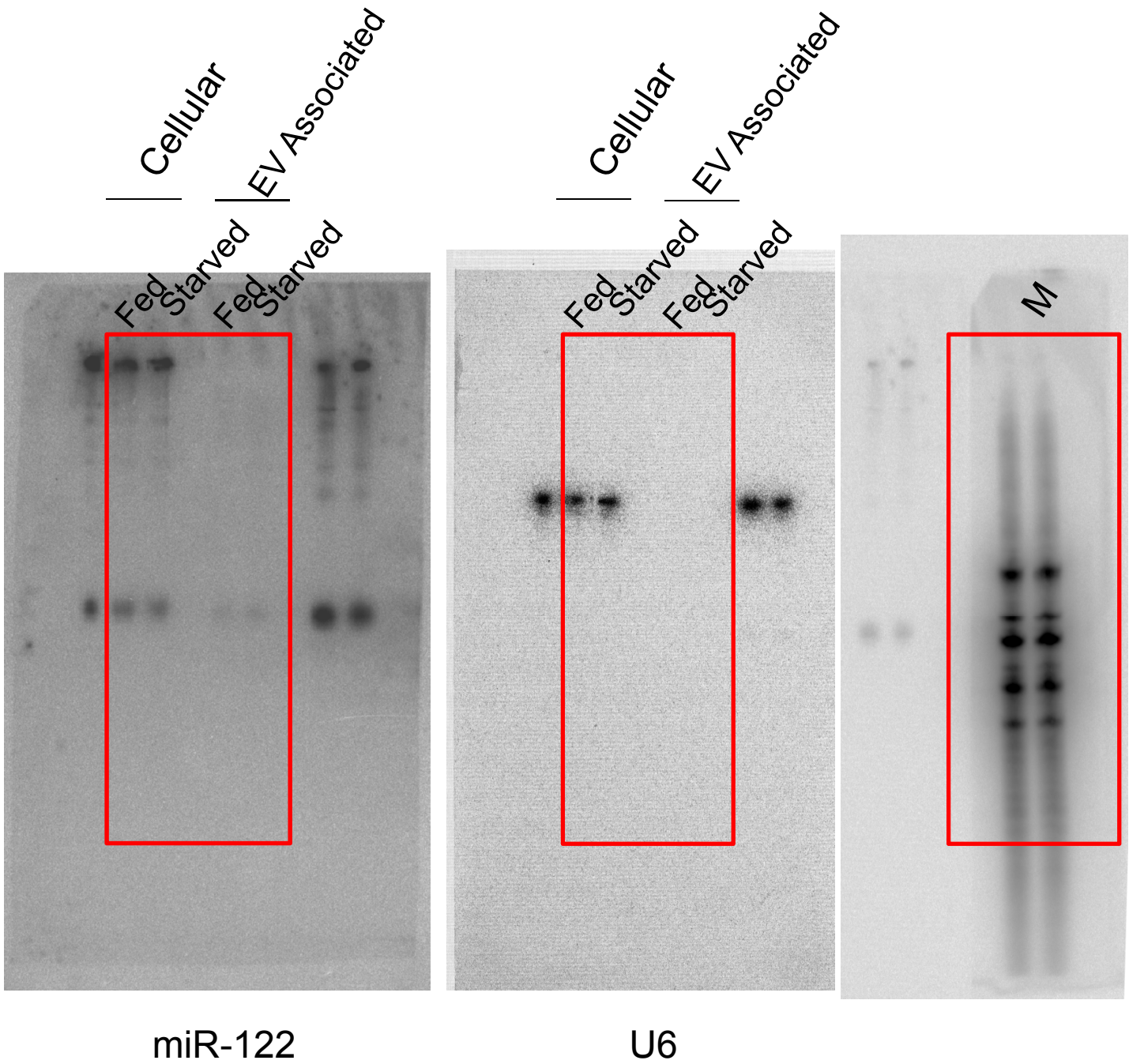

Supplement: Supplementary file 6 — Source Data for Figure 1A [file EMBR-17-1184-s005.pdf]

**Source Data Figure 5E**

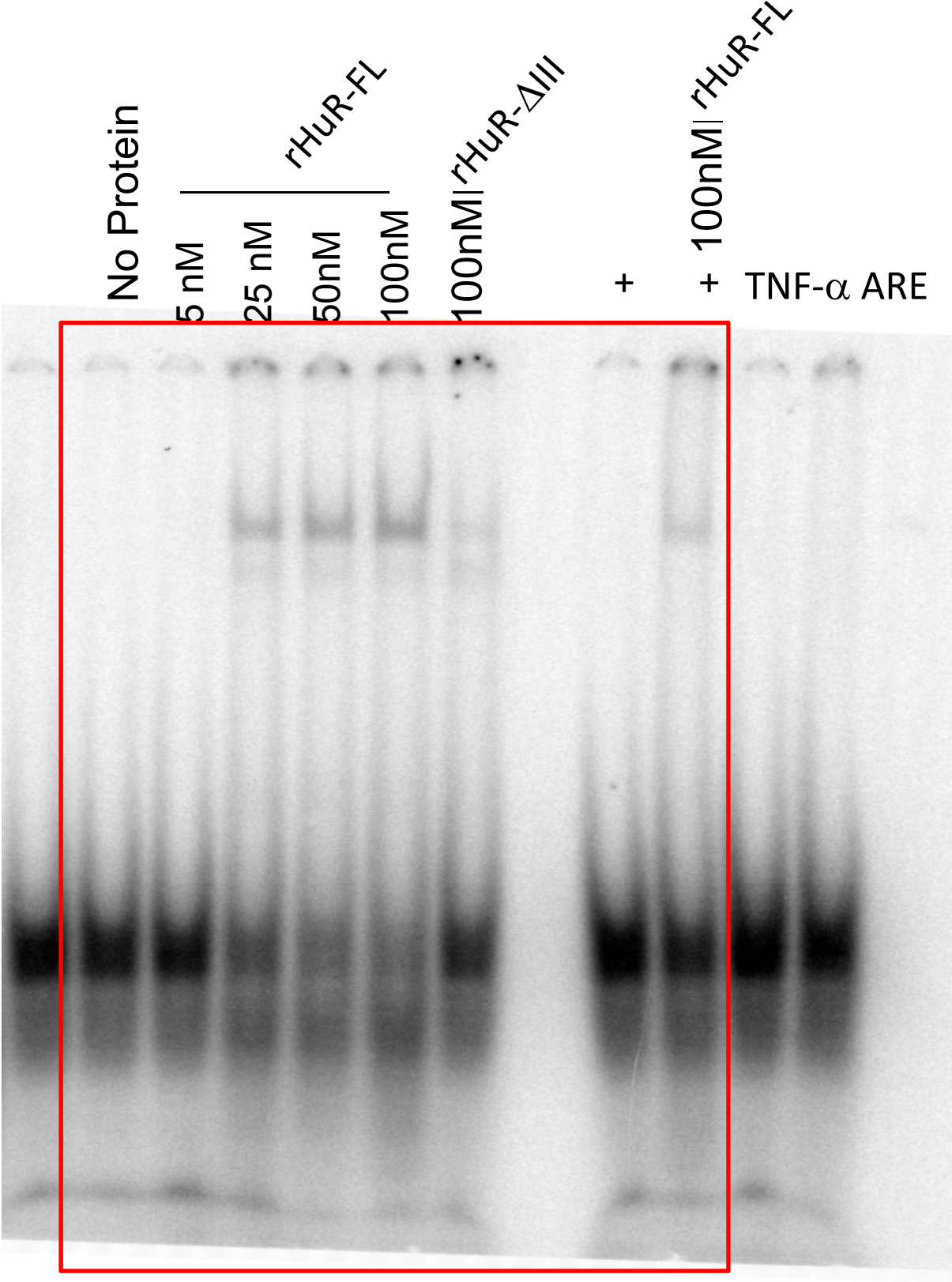

Supplement: Supplementary file 7 — Source Data for Figure 5E [file EMBR-17-1184-s006.pdf]

Source Data Figure 6J

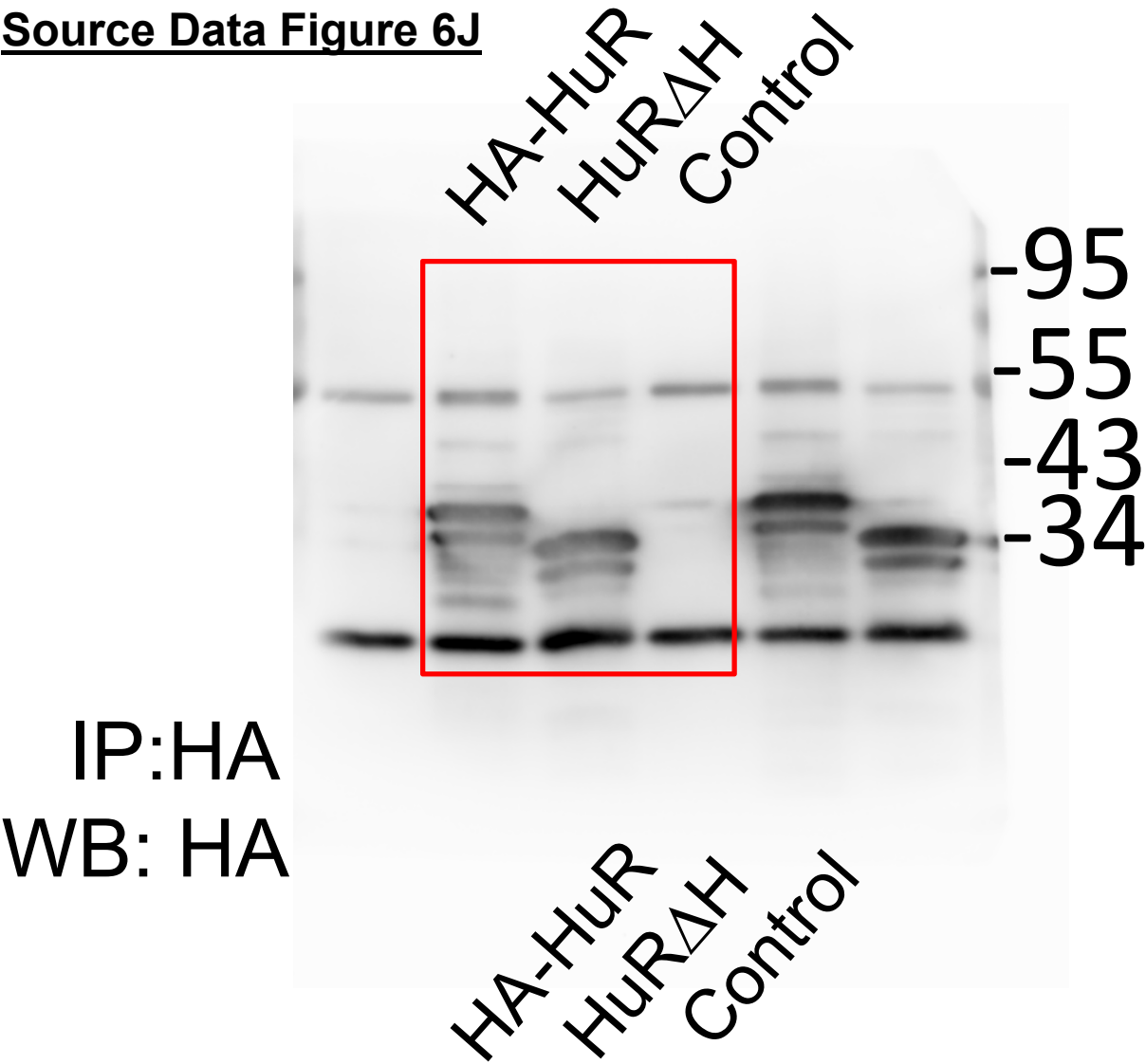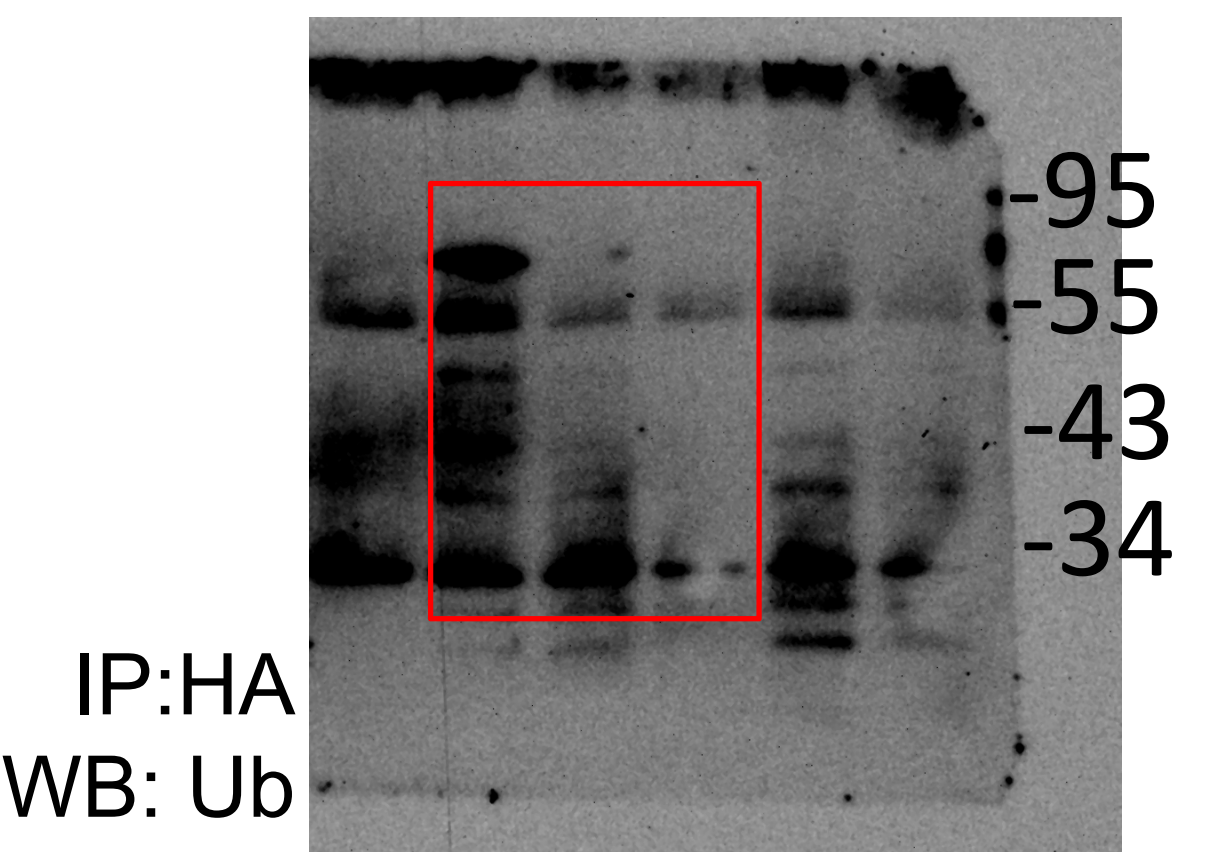

Supplement: Supplementary file 8 — Source Data for Figure 6 [file EMBR-17-1184-s007.zip › Source_Data_Fig_6J.pdf]

**Source Data Figure 6D**

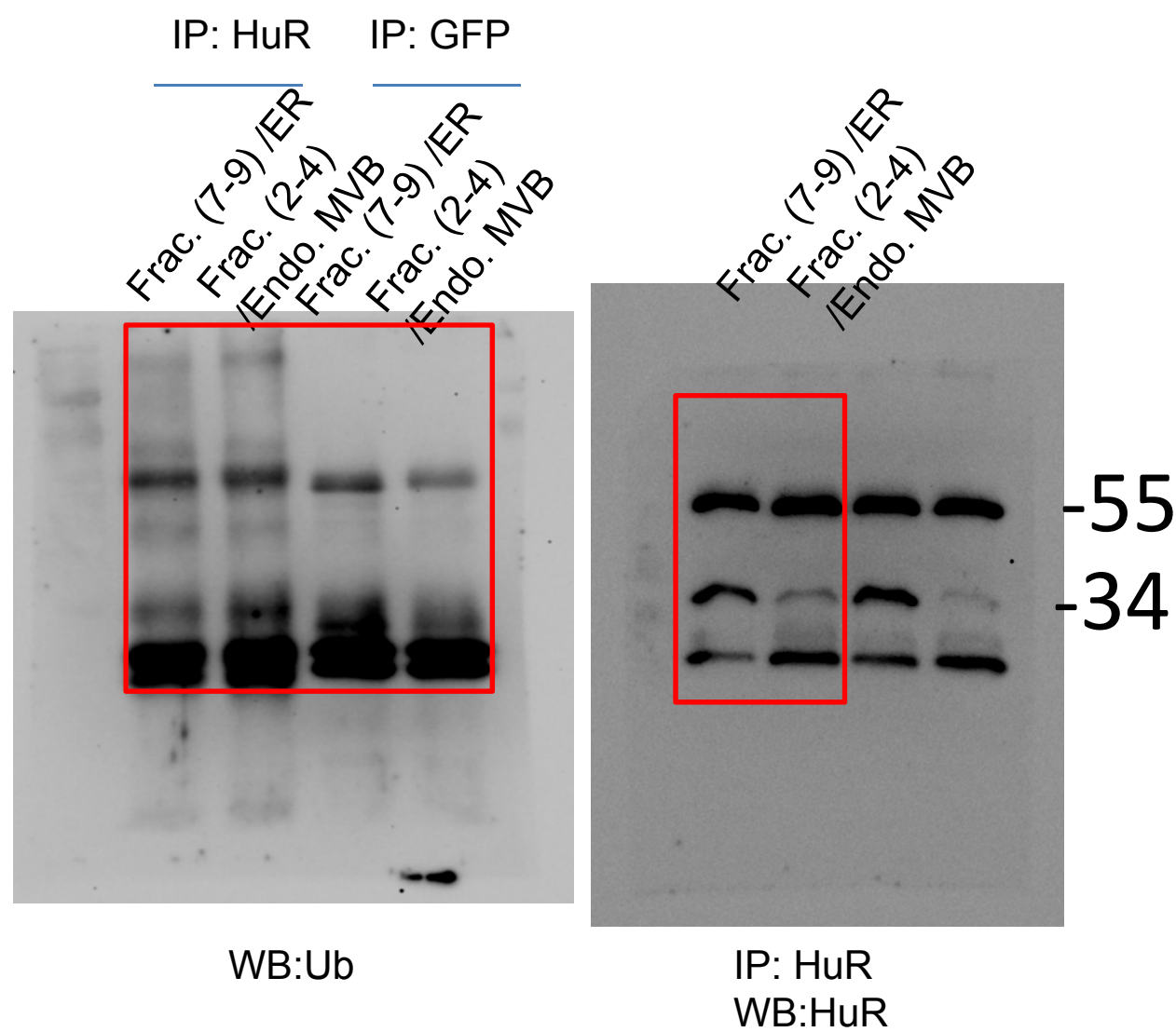

Supplement: Supplementary file 8 — Source Data for Figure 6 [file EMBR-17-1184-s007.zip › Source_Data_Fig_6D.pdf]

Source Data Figure 6G

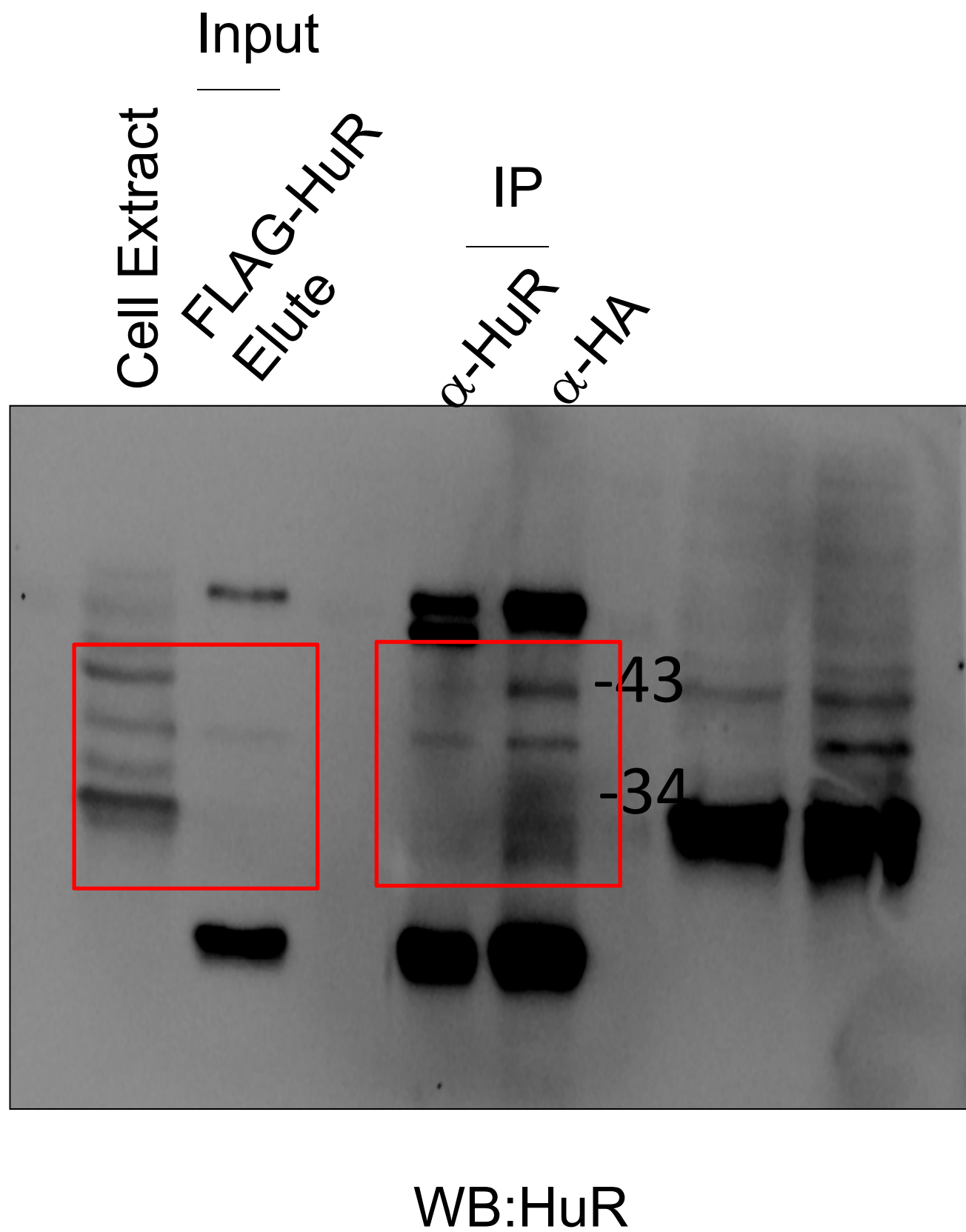

Supplement: Supplementary file 8 — Source Data for Figure 6 [file EMBR-17-1184-s007.zip › Source_Data_Fig_6G.pdf]
